# Advances in Structural Virology via Cryo-EM in 2022

**DOI:** 10.3390/v15061315

**Published:** 2023-06-02

**Authors:** Guy Schoehn, Florian Chenavier, Thibaut Crépin

**Affiliations:** Institut de Biologie Structurale (IBS), CEA, CNRS, Université Grenoble Alpes, 38058 Grenoble, France; guy.schoehn@ibs.fr (G.S.); florian.chenavier@ibs.fr (F.C.)

In recent years, cryo-electron microscopy (cryo-EM) has emerged as an important standalone technique within structural biology. The resolution revolution, which resulted in the award of the 2017 Nobel Prize in Chemistry [1,2], has made it possible to reach near-atomic resolutions with technological advances in instrumentation (in not only microscopes but also cameras with direct electron detectors); advanced automation; freezing conditions with more reproducible control, allowing finer ice to be obtained; and, of course, image analysis, particularly in the application of maximum likelihood principles. This combination of high-quality images coupled with advanced software running on powerful computers allows new questions to be answered. In addition to improving analyses of classical images, the current software within cryo-electron microscopy enables the separation of multiple conformations that can be adopted by a macromolecular complex [3] and even the separation of different macromolecular species present in a sample [4]. If the obtained resolution is still not optimal, the experimental electron density maps can then be combined with three-dimensional (3D) models obtained using structure prediction software such as AlphaFold2 [5,6]. Much progress has also been made in the production and purification of biological samples, which is a primary technique for obtaining high-quality structures.

These advances in structural biology (cryo-EM image analysis and AlphaFold2 structure prediction) are well adapted to structural virology. Viral particles are often difficult to crystallize. This particularly applies to particles that can assemble according to various triangulation numbers, such as enveloped alphaviruses (RNA viruses). For instance, Kaelber et al. confirmed that Eastern equine encephalitis virus (EEEV) particles can adopt T = 3 or T = 4 triangulation-based architectures. The separation of the structures can be performed in silico from images collected on a mixture of species. The T = 3 form represents a promising tool for designing alphavirus-based particles that deliver RNA to a specific target [7]. Bacteriophages are also challenging objects for crystallization with their large icosahedral-capsid-harboring tails of varying length and flexibility. In the case of podovirus GP4-infecting *Ralstonia solanacearum*, Zheng et al. showed that the capsid was composed of 540 copies of the major capsid protein (MCP) gp2 and 540 copies of the cement protein (CP) gp1, which were arranged in an icosahedral shell with a triangulation number of T = 9. The obtained resolution (3.7 Å) showed that the structures of gp2 and gp1 have a canonical HK97-like fold and an Ig-like fold, respectively. This new structure grants (and will continue to grant in the future) a better understanding of *Ralstonia solanacearum* infections by bacteriophages [8]. Wang et al. described the structure of the algal viruses of the *Marnaviridae* family. More precisely, they presented the structures of both the empty and full capsids of the *Chaetoceros socialis* forma radians RNA virus. By combining the structures of other *Picornaviridae* and the structure predictions produced with AlphaFold2, they succeeded in subclassifying the *Marnaviridae* according to their host at the level of host-specific receptor-binding mechanisms [9].

Beyond the structure of complete viral particles, cryo-EM can also be used in a more focused way to detail the attachment mechanisms of viruses to cells or to understand the plasticity and stabilization of surface glycoproteins. Pedenko et al. offer perspectives on the field both before and after the COVID-19 pandemic, with a particular focus on the SARS-CoV-2 glycoprotein. The different strategies of the prefusion conformation stabilization of the SARS-CoV-2 spike protein (S) are also discussed. The importance of structure-based approaches is highlighted by a comparison of the protective efficacy of stabilized and non-stabilized S trimers in vaccines [10]. Indeed, the vast majority of SARS-CoV-2 S structures have been determined by cryo-EM.

The application of cryo-EM in structural virology can explain the internal components of viruses and their operation modes in the context of viral replication. This is considered by Hutin et al., who analyze the helicase of the vaccinia virus (poxvirus). By combining advanced cryo-EM and AlphaFold2 prediction, they obtained the structure of the complex at pseudo-atomic resolution and tackled the problem of the existing flexibility between the primase and helicase parts of the pentameric D5 complex [11]. In recent years, cryo-EM has had the most impressive impact on the field of RNA virus replication and on molecular mechanisms. As highlighted in the review conducted by Ramaswamy et al., cryo-EM has emerged as a powerful tool for unraveling the structural heterogeneity of RdRps at the atomic level, which results from the interaction of various factors such as nucleic acids, cofactors or inhibitors [12]. Similarly, there has been a considerable expansion in the number of nucleocapsid structures, which has helped to explain viral genome encapsidation, as elegantly summarized in [13]. Genome packaging is highly studied for bacteriophages and herpesviruses and plays a pivotal role in protecting, transporting and facilitating genetic exchange. Lokareddy et al. reviewed the present understanding of viral small terminase’s involvement in genome packaging. TerS has been highly studied since its discovery 40 years ago, but its role in DNA packaging is still not clearly understood. Cryo-EM may be the solution for capturing a snapshot of this protein with its partner and, thus, for discovering the involvement of TerS in genome packaging [14].

Finally, the last article of our Special Issue concerns Nodaviruses, with a focus on multifunctional protein A, the key protein required for Nodavirus RNA replication crown formation, which is contiguous to the outer mitochondrial membrane. The obtained structures have significant implications for crown structure, assembly, function and control as an antiviral target [15]. This shows the power of cryo-electron tomography, which is undoubtedly the future of integrative structural biology and will be increasingly applied to the study of viruses and their life cycles in the future.
